# Identifying the primary tumour in patients with cancer of unknown primary (CUP) using [^18^F]FDG PET/CT: a systematic review and individual patient data meta-analysis

**DOI:** 10.1007/s00259-024-06860-1

**Published:** 2024-08-14

**Authors:** Jeroen R. J. Willemse, Doenja M. J. Lambregts, Sara Balduzzi, Winnie Schats, Petur Snaebjornsson, Serena Marchetti, Marieke A. Vollebergh, Larissa W. van Golen, Zing Cheung, Wouter V. Vogel, Zuhir Bodalal, Sajjad Rostami, Oke Gerke, Tharani Sivakumaran, Regina G.H. Beets-Tan, Max J. Lahaye

**Affiliations:** 1 Department of Radiology, the Netherlands Cancer Institute, Plesmanlaan 121, Amsterdam, 1066CX The Netherlands; 2 GROW Research Institute for Oncology and Reproduction – Maastricht University, Maastricht, Netherlands; 3 Department of Biometrics, Netherlands Cancer Institute, Amsterdam, Netherlands; 4 Department of Scientific Information Service, Netherlands Cancer Institute, Amsterdam, Netherlands; 5 Department of Pathology, Netherlands Cancer Institute, Amsterdam, Netherlands; 6 Faculty of Medicine, University of Iceland, Reykjavik, Iceland; 7 Department of Medical Oncology and Clinical Pharmacology, Netherlands Cancer Institute, Amsterdam, Netherlands; 8 Department of Gastrointestinal Oncology, Netherlands Cancer Institute, Amsterdam, Netherlands; 9 Department of Nuclear Medicine, Netherlands Cancer Institute, Amsterdam, Netherlands; 10 Department of Radiation Oncology, Netherlands Cancer Institute, Amsterdam, Netherlands; 11 Department of Clinical Research, University of Southern Denmark, Odense, Denmark; 12 Department of Nuclear Medicine, Odense University Hospital, Odense, Denmark; 13 Department of Medical Oncology, Peter MacCallum Cancer Centre, Melbourne, VIC Australia; 14 Sir Peter MacCallum Department of Oncology, University of Melbourne VIC, Melbourne, VIC Australia; 15 Faculty of Health Sciences, University of Southern Denmark, Odense, Denmark

**Keywords:** Cancer of unknown primary, [^18^F]FDG PET/CT, Oncology, Molecular imaging, Meta-analysis

## Abstract

**Purpose:**

In this systematic review and individual patient data (IPD) meta-analysis, we analysed the diagnostic performance of [^18^F]FDG PET/CT in detecting primary tumours in patients with CUP and evaluated whether the location of the predominant metastatic site influences the diagnostic performance.

**Methods:**

A systematic literature search from January 2005 to February 2024 was performed to identify articles describing the diagnostic performance of [^18^F]FDG PET/CT for primary tumour detection in CUP. Individual patient data retrieved from original articles or obtained from corresponding authors were grouped by the predominant metastatic site. The diagnostic performance of [^18^F]FDG PET/CT in detecting the underlying primary tumour was compared between predominant metastatic sites.

**Results:**

A total of 1865 patients from 32 studies were included. The largest subgroup included patients with predominant bone metastases (*n* = 622), followed by liver (*n* = 369), lymph node (*n* = 358), brain (*n* = 316), peritoneal (*n* = 70), lung (*n* = 67), and soft tissue (*n* = 23) metastases, leaving a small group of other/undefined metastases (*n* = 40). [^18^F]FDG PET/CT resulted in pooled detection rates to identify the primary tumour of 0.74 (for patients with predominant brain metastases), 0.54 (liver-predominant), 0.49 (bone-predominant), 0.46 (lung-predominant), 0.38 (peritoneal-predominant), 0.37 (lymph node-predominant), and 0.35 (soft-tissue-predominant).

**Conclusion:**

This individual patient data meta-analysis suggests that the ability of [^18^F]FDG PET/CT to identify the primary tumour in CUP depends on the distribution of metastatic sites. This finding emphasises the need for more tailored diagnostic approaches in different patient populations. In addition, alternative diagnostic tools, such as new PET tracers or whole-body (PET/)MRI, should be investigated.

**Supplementary Information:**

The online version contains supplementary material available at 10.1007/s00259-024-06860-1.

## Introduction

Cancer of unknown primary (CUP) can be defined as histologically confirmed metastatic cancer in which standard diagnostic methods do not identify a primary tumour [1]. Approximately 2–5% of cancer cases worldwide are estimated to be CUP [2]. Because patients with CUP have metastatic disease, survival rates are abysmal, with a median survival of 2–12 months [3]. Adenocarcinomas account for approximately 70–80% of CUP, whereas the rest are undifferentiated, squamous, and neuroendocrine carcinomas [4]. Autopsy studies conducted before 2010 have shown that CUP mainly originates from lung cancer (27%) and pancreatic or hepatobiliary cancer (24% and 8%, respectively) [5]. However, the available diagnostic armamentarium per time period will affect such data.

Identifying the primary tumour allows for standard-of-care treatment options, potential inclusion in trials for identified primary tumours, and possibly other targeted therapies with a consequent positive impact on survival [6, 7]. Establishing guidelines for the diagnostic work-up remains challenging given the heterogeneous nature of CUP patients. Guidelines from the European Society of Medical Oncology (ESMO) state that the minimal mandatory clinical work-up should consist of a thorough history and physical examination, basic blood analyses, and either computed tomography (CT) with an intravenous contrast agent or MRI scans of the head & neck, chest, abdomen and pelvis. Additional mammography is advised in women. Tissue sampling is required to allow histological and immunohistochemical analyses to guide the search for the underlying primary tumour [8]. If the routine diagnostic work-up fails to detect a primary tumour, tailored diagnostic strategies can be used, depending on patient characteristics, location of the metastases, and genetic profiling.

Whole-body 2-deoxy-2-[^18^F]fluoro-D-glucose Positron Emission Tomography/ Computed Tomography ( [^18^F]FDG PET/CT) is a frequently used second-line imaging technique after CT in CUP patients, as it can aid in identifying the primary tumour and depicting the true extent of the disease. The ESMO guidelines currently recommend the use of [^18^F]FDG PET/CT to rule out additional manifestations in patients with single-site/oligometastatic CUP or in patients with cervical metastases that are likely to originate from head and neck cancers, mostly squamous cell carcinomas, which are highly hypermetabolic [8]. In the latter group, a recent systematic review found that [^18^F]FDG PET/CT had a pooled detection rate of primary tumours of 40% [9].

Due to the heterogeneous nature of CUP, other studies investigating the potential additional role of [^18^F]FDG PET/CT have mainly included mixed patient cohorts with various metastatic locations, histopathological features, and primary tumours. Using aggregate data from heterogeneous study populations typically limits the applicability of conventional systematic reviews and meta-analyses, as these do not account for the large heterogeneity in included cohorts. At the same time, large numbers of heterogeneous study populations provide a unique opportunity to group individual patients from individual cohorts into larger subgroups. This allows for more specific analyses of the role of [^18^F]FDG PET/CT in different subgroups of CUP patients.

This study aimed to perform a retrospective individual patient data (IPD) meta-analysis of CUP patients to analyse the diagnostic performance of [^18^F]FDG PET/CT for primary tumour detection rate in relation to the most predominant metastatic site and to map the distribution of underlying primary tumours for each subgroup.

## Methods

This IPD systematic review and meta-analysis was conducted according to the Preferred Items for Systematic Reviews and Meta-Analyses of Individual Participant Data (PRISMA-IPD) [10] guidelines and was registered in the prospective systematic review database PROSPERO under registration number CRD42023401409.

### Literature search and inclusion criteria

A comprehensive search was conducted across multiple electronic databases (MEDLINE, EMBASE, SCOPUS) from 1 January 2005 to 14 February 2024 to identify all randomised controlled trials and retrospective cohort studies describing the diagnostic value of [^18^F]FDG PET/CT in CUP. The search strategy included the terms [^18^F]Fluorodeoxyglucose, cancer of unknown primary and relevant synonyms or abbreviations. The entire search strategy is shown in Online Resource 1.

The titles, abstracts, and complete text reports of the retrieved studies were screened for eligibility. We considered studies eligible for inclusion if CUP was defined as histologically or radiologically confirmed solid or mucinous metastatic cancer and standard diagnostic methods (including CT) did not identify a primary tumour. Studies focusing solely on cervical lymph node metastases to identify occult head and neck tumours were excluded as this subgroup has already been addressed in a recent meta-analysis by Huasong et al. [9]. The reference lists of all included papers were cross-checked for additional relevant studies. Authors of papers that did not report individual patient data and authors of conference proceedings were contacted with a data request. In the case of no response, a reminder was sent three weeks after the initial attempt. Studies were excluded if individual patient data could not be retrieved. If a study assessed both PET only and PET/CT, patients with PET only (without CT) were excluded.

### Quality assessment

The quality of all included published papers was assessed using the Quality Assessment of Diagnostic Accuracy Studies-2 (QUADAS-2) tool [11]. The signalling questions tailored to this review are shown in Online Resource 2A.

### Individual patient data analysis

Metastatic locations, primary tumour sites suggested by [^18^F]FDG PET/CT, and available reference standards were collected at the patient level. Individual patients were excluded from further analyses based on the following exclusion criteria: (a) patients with primarily cervical metastases; (b) patients not conforming to the definition of CUP as described in the previous section; and (c) patients who underwent PET only (without CT). The primary tumour diagnosis (if any) as established by [^18^F]FDG PET/CT was matched with the reference standard (established as described in the final column of Table 1) and classified as true positive (TP), false positive (FP), false negative (FN), or Confirmed CUP (no diagnosis), using the following definitions:

- *TP*: [^18^F]FDG PET/CT diagnosis of the primary tumour matched the final reference standard.
- *FP*: [^18^F]FDG PET/CT diagnosis of the primary tumour did not match the final reference standard.
- *FN*: no primary tumour was found on [^18^F]FDG PET/CT, but a primary tumour diagnosis was established using another method (e.g. colonoscopy, genomic profiling, etc.).
- *Confirmed CUP*: no primary tumour was found on [^18^F]FDG PET/CT nor using any other methods, or during further follow-up(no diagnosis).

We divided the patients into eight subgroups based on the predominant metastatic site: bone, brain, liver, lung, lymph nodes, peritoneal, soft tissue, and ‘other’ metastases. To measure the diagnostic accuracy of [^18^F]FDG PET/CT, we considered the primary tumour detection rates (DRs), defined as the number of TP out of the number of subjects included, per study. For each subgroup, pooled DRs, corresponding 95% confidence intervals (CI), and prediction intervals were calculated. The random effects model considering the inverse variance method and logit transformed proportions was applied. The I^2^-statistic and τ^2^ were used to assess heterogeneity. A Sankey diagram was used to visualise the relationship between metastatic sites and primary tumours. Data analysis was performed using R Studio (version 6.2) and meta-analyses were conducted using the *meta* package.

## Results

### Studies

**Fig. 1:**
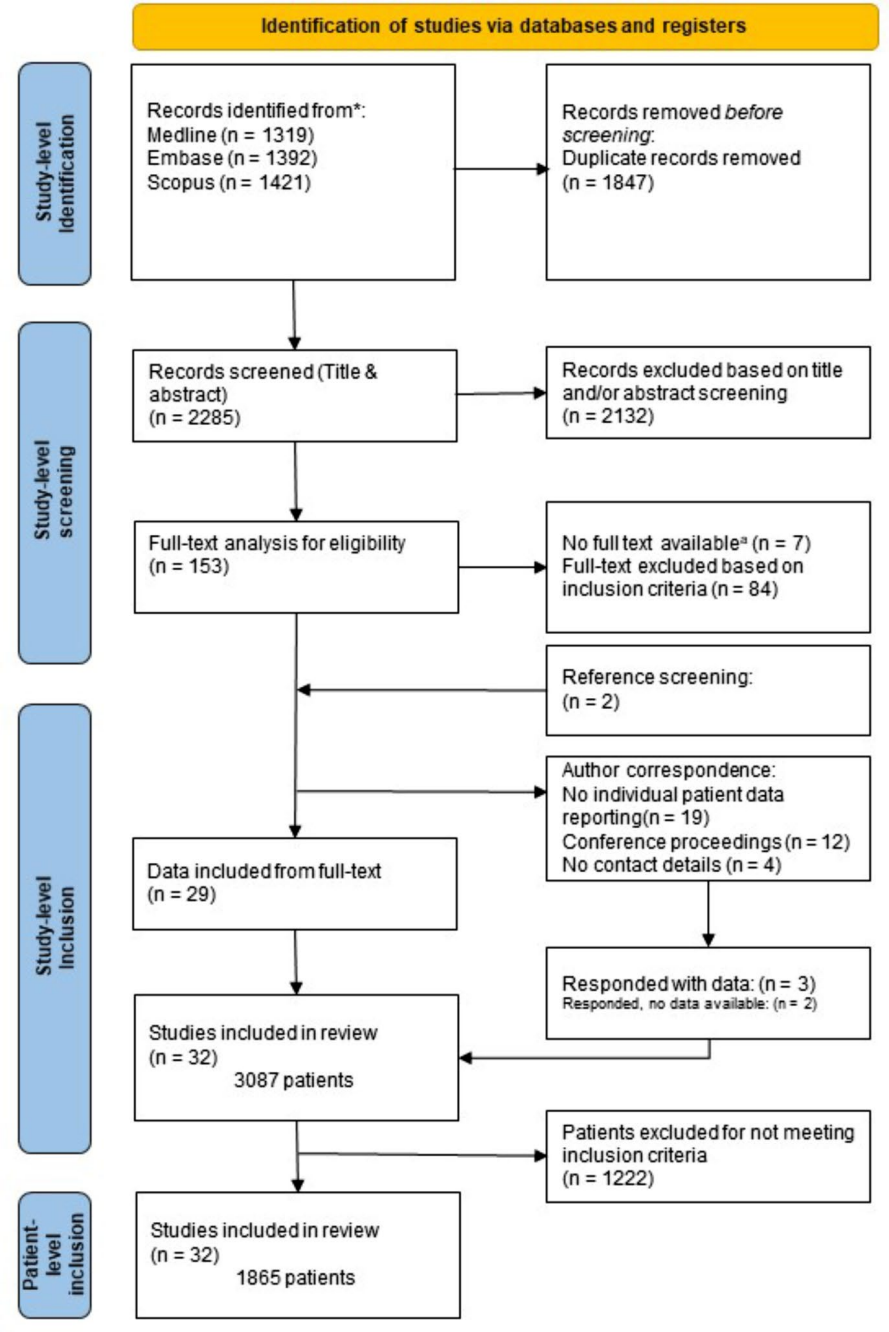
PRISMA flowchart depicting the process of identifying relevant studies and inclusion of patients. ^a^Excluding conference proceedings for which no full text is available by default

A total of 2285 unique studies were identified after searching Medline, Embase, and Scopus. Following title and abstract screening, 153 studies were assessed for full-text availability and eligibility for inclusion. Figure 1 shows the inclusion process, resulting in individual patient data of 1865 patients from 31 original studies and one conference proceeding. Table 1 shows the characteristics of the included studies. Two studies had partially overlapping study populations, which were corrected by excluding overlapping patients from one study [12, 13].

**Table 1:** Characteristics of included studies

Study	Study characteristics				Number of patients		[^18^F]FDG PET/CT		Reference standard	
	Author	Year	Country	Design	Original paper	Patients included in this review^a^	Activity(MBq)^b^	Uptake time(min)		
1	Ambrosini [14]	2006	Italy	NR	38	32	370	60–90	Histopathology & clinical FU	
2	Bicakci [15]	2022	Turkey	Retrospective	125	92	250–370	NR	Histopathology & clinical FU	
3	Budak [16]	2020	Turkey	Retrospective	100	98	259–407	60	Histopathology & clinical FU	
4	Cengiz [17]	2018	Turkey	Retrospective	121	93	270–370	60	Histopathology & clinical FU	
5	Deonarine [18]	2013	Scotland	Retrospective	51	36	350–400	60	Histopathology & clinical FU	
6	Fencl [19]	2007	Czech Republic	Retrospective	190	62	350–450	60–90	Histopathology & clinical FU	
7	Gutzeit [20]	2005	Germany	Retrospective	45	27	350	60	Histopathology	
8	Jain [21]	2015	India	Prospective	86	86	185–245	60	Histopathology & clinical FU	
9	Koc [22]	2018	Turkey	Retrospective	31	26	370	NR	Histopathology	
10	Lawrenz [23]	2020	U.S.A.	Retrospective	35	13	NR	NR	NR	
11	Lee [24]	2012	Hong Kong	Retrospective	58	39	370	45–50	Histopathology & clinical FU	
12	Li [25]	2020	China	Retrospective	124	122	259	45–60	Histopathology & clinical FU	
13	Mohamed [26]	2021	Egypt	Prospective	39	39	175	60	Histopathology & clinical FU	
14	Nanni [27]	2005	Italy	Prospective	18	17	370	60–90	Histopathology	
15	Nikolova [28]	2021	Bulgaria	Retrospective	53	14	270–370	60	Histopathology & clinical FU	
16	Ora [29]	2022	India	Retrospective	50	24	259	60–90	Histopathology & PET/CT appearance	
17	Ozkan [30]	2016	Turkey	Retrospective	37	25	555	60	Histopathology & clinical FU	
18	Park, J.S [31].	2011	Korea	Retrospective	20	10	555–740	60–90	Histopathology	
19	Park, S.B [32].	2018	Korea	Retrospective	103	85	385	60	Histopathology & clinical FU	
20	Pelosi [33]	2006	Italy	Retrospective	68	40	222–370	60	Histopathology & clinical FU	
21	Rimer [34]	2023	Denmark	Retrospective	159	106	280	45–75	Histopathology & clinical FU	
22	Saidha [35]	2013	India	Prospective	50	34	350–425	60	Histopathology	
23	Sivakumaran [36]	-	Australia	Retrospective	147	93	252	60–75	Histopathology & clinical FU	
24	Soni [37]	2021	U.S.A.	Retrospective	83	63	260–600	60–90	Histopathology & clinical FU	
25	Tamam (2012) [12]	2012	Turkey	Retrospective	316	143	296–555	60	Histopathology & clinical FU	
26	Tamam (2016) [13]	2016	Turkey	Retrospective	87	87	296–555	50	Histopathology & clinical FU	
27	Wang [38]	2013	China	Retrospective	142	78	270–370	60–75	Histopathology & clinical FU	
28	Wolpert [39]	2018	Switzerland	Retrospective	64	64	NR	NR	Histopathology & clinical FU	
29	Yapar [40]	2010	Turkey	NR	94	49	222–370	60–90	Histopathology & clinical FU	
30	Yoo [41]	2020	Korea	Retrospective	74	51	385	50	Histopathology & clinical FU	
31	Yu [42]	2016	China	Retrospective	449	99	282–330	60	Histopathology & clinical FU	
32	Zidan [43]	2020	Egypt	Retrospective	30	18	259	45–90	Clinical & radiological FU	

^a^ Individual patients from the original study cohorts may have been excluded according to our exclusion criteria. ^b^ In the case of reported administered doses per kilogram, an average body weight of 70 kg was assumed. *NR* not reported; *MBq* megabecquerel; *FU* follow-up; *min* minute

### Quality assessment

**Fig. 2:**
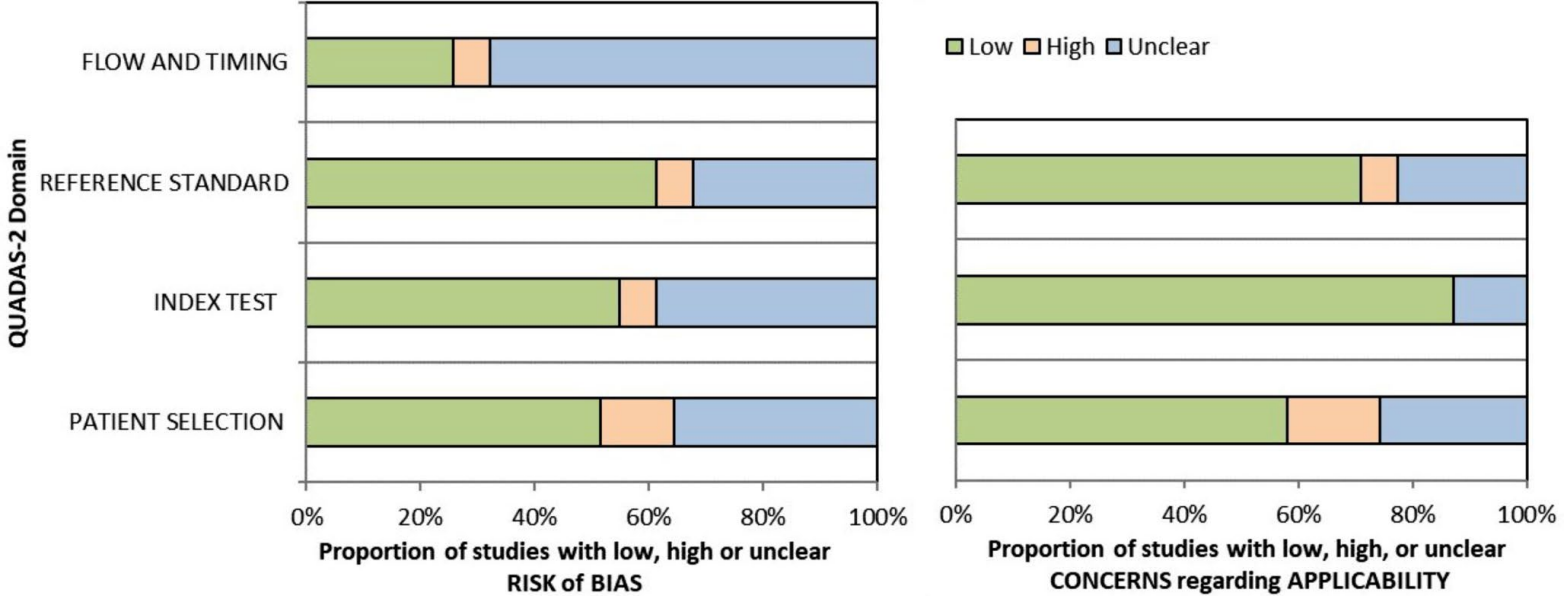
QUADAS-2 results: an overview of 31 studies with full-text availability

Thirty-one of thirty-two studies had full-text availability. One conference proceeding was included and could not be assessed using the QUADAS-2 checklist [11]. The results are shown in Fig. 2. The full QUADAS-2 assessment on a per-study basis is shown in Online Resource 2B.

### Patients

In total, 1865 patients from 32 studies were included in this analysis. The number of patients included in each study ranged from 10 to 143, with a median of 50 patients. In one study, only a part of the population underwent PET/CT, whereas the remaining participants were excluded because they underwent PET only [31]. The characteristics of the included patients are shown in Table 2. The diagnostic workup that patients underwent prior to PET/CT was poorly documented in the majority of patients. In nine studies (460 patients), it was explicitly reported that patients had undergone an extensive diagnostic workup prior to [^18^F]FDG PET/CT, consisting of at least a CT scan, laboratory, and physical examinations. In the remaining studies, the extent of the pre-PET/CT diagnostic work-up was either not well defined or not reported (1405 patients).

In most studies, the reference standard for determining the primary tumour was established by histopathology and/or clinical follow-up. In the case of positive findings, discovered lesions were typically biopsied to obtain histological confirmation, while in the case of negative findings, clinical follow-up was implemented as the main standard of reference to establish a definitive negative (CUP) diagnosis.

**Table 2:** Distribution of 1865 included patients according to predominant metastatic site and final primary tumour diagnosis

Total	1865	
**Predominant metastatic site**	**#**	**(%)**
Bone	622	33%
Liver	369	20%
Lymph nodes	358	19%
*Thoracic*	164	9%
*Abdominopelvic*	102	5%
*Non-specified*	92	5%
Brain	316	17%
Peritoneum	70	4%
Lung	67	4%
Soft tissue	23	1%
Other	40	2%
**Final primary tumour diagnosis**	**#**	**(%)**
Confirmed CUP	607	33%
Lung/ bronchi	546	29%
Colorectum	101	5%
Esophagus/ stomach	77	4%
Breast	69	4%
Other/ non-specified	59	3%
Prostate	57	3%
Pancreas	54	3%
Gynecological organs	54	3%
Bile duct	46	2%
kidney/ urothelial tract	36	2%
Lymphoma	37	2%
Bone/ sarcoma	31	2%
Head & neck/ thyroid	31	2%
Liver	22	1%
Melanoma/ skin	13	1%
Brain	12	1%
Small bowel	13	1%

### Detection rates of the primary tumour in relation to the predominant metastatic site

**Fig. 3:**
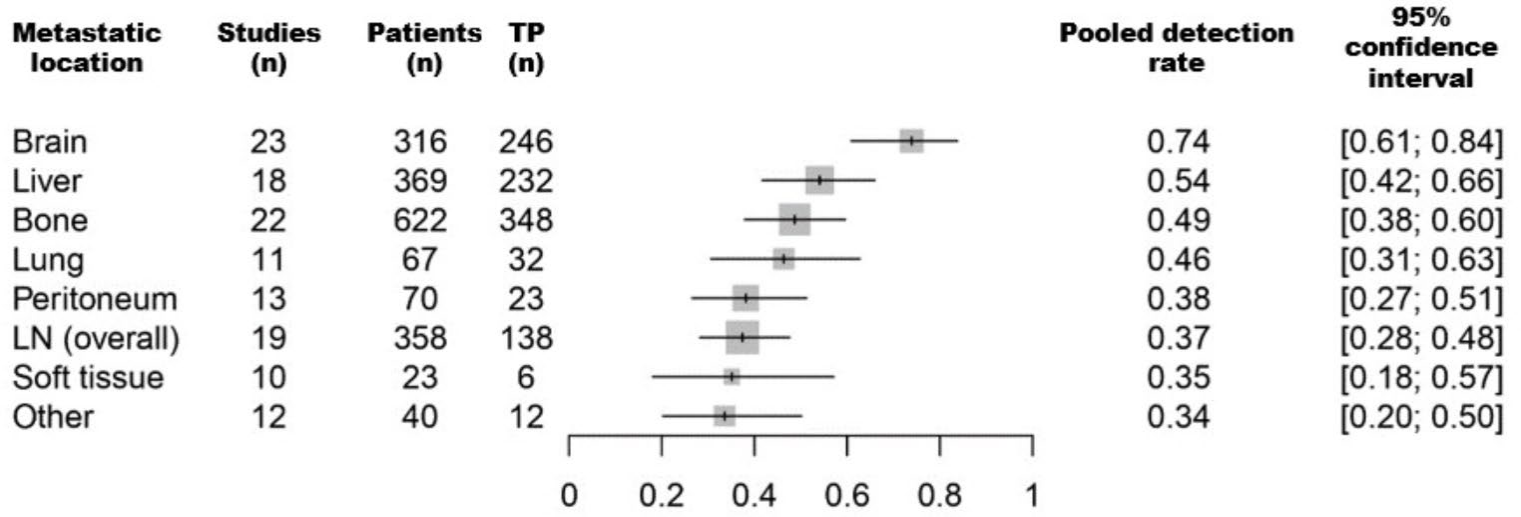
Forest plot of pooled detection rates of [^18^F]FDG PET/CT across different patient subgroups according to the most predominant metastatic site. *LN* lymph nodes; *TP* true positives

The pooled DRs of primary tumours varied between 0.74 (for patients with predominant brain metastases) and 0.35 (for patients with soft tissue metastases). Figure 3 shows a forest plot of the pooled DRs for each subgroup. Overall, a pooled detection rate of 0.54 (CI: [0.45; 0.64]) was found.

Table 3 provides an overview of the [^18^F]FDG PET/CT results per predominant metastatic site group, including a specification of the types of primary tumour diagnoses. Overall (in 6/10 subgroups) lung cancer was the most frequently detected primary tumour, diagnosed in 29% of our total study population. The second and third most common primary tumour types were colorectal cancer (*n* = 101; 5%) and oesophageal and gastric cancers (*n* = 77; 4%). In the final columns, the heterogeneity of the results across the different studies is shown for each subgroup. A more detailed table of all primary tumours and corresponding classifications (TP/FP/FN/Confirmed CUP) is shown in in Online Resource 3.

**Table 3:** Classification of [^18^F]FDG PET/CT findings (TP/FP/FN/CUP) and most common final diagnoses per subgroup. The final two columns show the measures of heterogeneity found in each subgroup

Metastatic Site	Total	[^18^F]FDG PET/CT Classification				True primary tumours		Random effects model	
		**TP**	**FP**	**FN**	**CUP**	**Confirmed true primary**	**Confirmed CUP**	**τ** ^**2**^	**I** ^**2**^
Brain	316	246 (78%)	11 (3%)	6 (2%)	53 (17%)	*Lung: 193*,* brain: 13*,* esophagus/stomach: 7*	61 (19%)	1.1061	67%
Liver	369	232 (63%)	23 (6%)	29 (8%)	85 (23%)	*Colorectum: 59*,* lung: 58*,* esophagus/stomach: 36*	93 (25%)	0.6425	71%
Lung	67	32 (48%)	6 (9%)	10 (15%)	19 (28%)	*Lung: 21*,* CRC: 7*	25 (37%)	0.2713	27%
Bone	622	348 (56%)	37 (6%)	77 (12%)	160 (26%)	*Lung: 216*,* prostate: 50*,* breast: 25*	180 (29%)	0.7319	76%
Soft tissue	23	6 (26%)	3 (13%)	4 (17%)	10 (43%)	*Lung: 3*,* kidney/urology: 2. breast: 2*	12 (52%)	0	0%
Peritoneum	70	23 (33%)	1 (1%)	5 (7%)	41 (59%)	*Colorectum: 9*,* gynecology: 7*,* esophagus/stomach : 6*	42 (60%)	< 0.0001	4%
Other	40	12 (30%)	6 (15%)	4 (10%)	18 (45%)	*Lung: 6*,* gynecology: 3*	21 (53%)	0.012	0%
Thoracic LN	164	64 (39%)	4 (2%)	21 (13%)	75 (46%)	*Lung: 33*,* breast: 23*,* lymphoma: 7*	78 (48%)	0.4952	52%
Abdominopelvic LN	102	34 (33%)	6 (6%)	10 (10%)	52 (51%)	*Lymphoma: 9*,* pancreas: 6*,* lung: 5*	55 (54%)	0.5317	35%
Not specified LN	92	40 (43%)	20 (22%)	4 (4%)	28 (30%)	*Gynecology: 12*,* lung: 11*,* lymphoma: 4*	40 (43%)	1.6780	77%
Total	1865	1037(56%)	117(6%)	170(9%)	541(29%)	*Lung: 546*,* colorectum: 101*,* esophagus/stomach: 77*	607 (33%)	*1.0025*	*90%*

*LN* Lymph nodes; *TP* True positive; *FP* False positive; *FN* False negative; *τ*^*2*^ Variance of true effect sizes between studies ; *I*^*2*^ Percentage of total variability due to heterogeneity

### Primary tumours

**Fig. 4:**
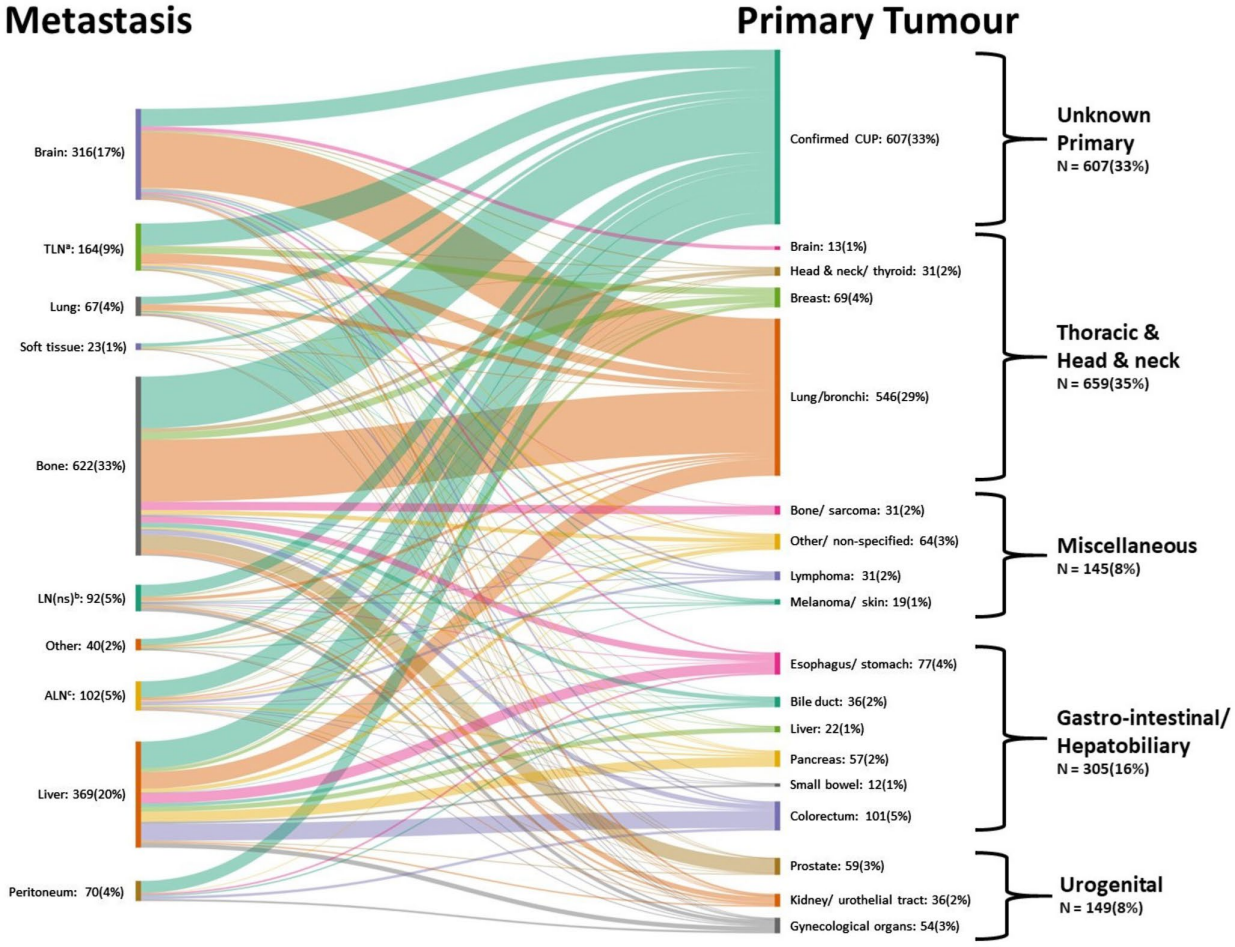
Sankey diagram: patients were grouped by metastatic sites on the left side. Flows and their relative sizes represent connections to the final diagnosis after [^18^F]FDG PET/CT and follow-up (true primary tumours or confirmed CUP). ^**a**^Thoracic lymph nodes; ^**b**^lymph nodes (not specified); ^**c**^abdominopelvic lymph nodes. Figure created using https://sankeymatic.com/

Overall, [^18^F]FDG PET/CT and follow-up revealed a primary tumour in 1258 out of 1865 patients (67%). In 1037 of these cases, the primary tumour was correctly identified by [^18^F]FDG PET/CT (pooled DR: 0.54), while in the remainder, follow-up revealed the primary tumour. Six hundred and seven (33%) patients were classified as having a confirmed CUP. Figure 4 presents a Sankey diagram visualising the corresponding primary tumours for each subgroup.

## Discussion

This review and meta-analysis of 1865 individual patient cases found an overall detection rate of 0.54 for [^18^F]FDG PET/CT in identifying primary tumours in patients with CUP. Interestingly detection rates varied considerably depending on the most predominant metastatic site, ranging from only 0.35–0.38 in patients with predominant soft tissue, lymph node and peritoneal metastases to as high as 0.74 in patients with predominant brain metastases. Detection rates for patients presenting primarily with bone, liver and lung metastases were in the intermediate range (0.46–0.54).

In 2017, a systematic review on the diagnostic performance of [^18^F]FDG PET/CT in CUP by Burglin et al. [44] found a lower detection rate (0.41) than the pooled overall detection rate (0.54) we found in our current review. This discrepancy might be caused by the included studies: three out of four studies with the lowest detection rates in the previous review were not included in our current study, as these studies did not provide sufficient data for the per-metastasis analysis performed in our review [45–47].

Lung tumours constituted the largest subgroup of primary tumour diagnoses by far, comprising 29% of the cohort. It is known from literature that primary lung cancers are the leading cause of CUP, accounting for an estimated 27% of primary tumours [48].

Interestingly, our cohort had a significantly smaller number of primary pancreatic or hepatobiliary primary tumours than expected based on autopsy and genetic profiling studies [48]. Although it is difficult to draw firm conclusions, one hypothesis could be that [^18^F]FDG PET/CT has a relatively limited performance in detecting abdominal primary tumours. For instance, in peritoneal metastases, which are generally likely to originate from abdominal primary tumours, [^18^F]FDG PET/CT could be a suboptimal imaging modality given its relatively low detection rate of primary tumours in this subgroup [49]. The limited soft-tissue contrast of low-dose CT could limit its ability to distinguish peritoneal disease from the adjacent abdominal organs from which the cancer is disseminated. Similarly, abdominal (low-volume) primary cancers might be easily missed by [^18^F]FDG PET/CT because of low spatial resolution and partial volume effects or get lost in physiological FDG-uptake as observed in the colon or urological system. In addition, primary tumours with low FDG avidity, such as mucinous, signet ring cell, or low grade neuroendocrine cancers, can be missed more easily by [^18^F]FDG PET/CT, as these are not hypermetabolic [50–52]. In other patients with non-hypermetabolic metastases, identification of the primary tumour through [^18^F]FDG PET/CT may also pose a challenge, as this is an indication of primary tumours with slower metabolism. In addition, high glucose levels decrease the sensitivity of [^18^F]FDG PET/CT in general and should be taken into account when considering [^18^F]FDG PET/CT.

Current ESMO guidelines state the use of MRI as an alternative modality to CT in searching for a primary tumour, even though research on MRI in the setting of CUP is limited [8]. Therefore, in addition to identifying CUP patients that might benefit from [^18^F]FDG PET/CT, the potential role of MRI should also be investigated in future research. For instance, whole-body diffusion-weighted MRI (WB-DWI/MRI) has shown promising diagnostic accuracy in different types of gastrointestinal cancer and could be considered a diagnostic imaging modality for patients with metastases that are likely to originate from the abdomen or pelvis [53–56].

By categorising patients according to the predominant metastatic site, we aimed to provide insight into which patients with CUP might benefit the most from [^18^F]FDG PET/CT. The differences in detection rates observed across subgroups further highlight the heterogeneity of CUP patients and oppose the idea of a ‘one-size-fits-all’ diagnostic approach. As outlined above, our results suggest that [^18^F]FDG PET/CT might not be the most suitable imaging technique for each CUP patient, and that further tailoring of imaging according to the pattern of disease could be beneficial.

Novel imaging strategies should also be considered as potential diagnostic tools for CUP. As newer, whole-body PET/CT systems are now available, which will result in a better image quality and higher diagnostic accuracy (when not compensating for the better quality by decreasing the injected activity), while imaging the whole body including the legs, in only five minutes [57]. In addition, novel tracers, e.g. radiolabeled FAPI (Fibroblast Activation Protein Inhibitor), might be superior to [^18^F]FDG in the detection of primary non-hypermetabolic tumours, like gastric, colorectal, biliary, hepatic and pancreatic cancer [58].

Apart from imaging, molecular(genetic) profiling techniques are emerging that could be of additional value in identifying the primary tumour in CUP patients. Over the past few years, whole-genome sequencing (WGS) has gained popularity in clinical practice. Using an algorithm to generate profiles indicative of specific tumour types by genetic profiling of tissue samples taken from biopsies, WGS can point to a specific primary tumour [59]. The integration of different new diagnostic modalities, such as whole-body MRI and WGS algorithms, combined with a structural multidisciplinary approach, might further improve the diagnostic work-up of CUP patients.

This study has some limitations. First, as is common in CUP research, establishing consistent and valid reference standards is a major challenge, and it is difficult to evaluate the validity of the reference standards used in the individual studies included in this review. Likewise, it was difficult to determine the risk of bias in the included studies. Since CUP is not a clearly defined diagnosis but rather a diagnosis per exclusionem, patients who eventually enrol in studies such as those included in this review have experienced different diagnostic investigations and time paths during their disease course. Other variations between the included studies that limit the generalisability of our results include differences in the pre-[^18^F]FDG PET/CT diagnostic workup and therefore the availability of clinicopathological information at the time of PET/CT evaluation, as well as varying [^18^F]FDG PET/CT procedures (including patient preparation, blood glucose levels at the time of acquisition and field of view) and improvements in scanner systems, in terms of hardware and software throughout the years. Additionally, there was limited information available on the definition of predominant metastatic disease and the possible presence of metastatic sites in addition to the single site presented in the included articles, restricting this review to containing analyses of single site metastatic disease only. Finally, some subgroups in our study were relatively small, limiting the applicability of these results.

## Conclusion

This individual patient data meta-analysis demonstrated that the detection rate of [^18^F]FDG PET/CT to identify the primary tumour in patients with CUP depends on the distribution of metastatic sites. Future studies should focus on exploring the potential of new diagnostic tools, including novel PET tracers, whole-body (PET/)MRI, and on further tailoring diagnostic strategies based on the predominant pattern of disease.

## Electronic supplementary material

Below is the link to the electronic supplementary material.

Supplementary Material 1

Supplementary Material 2

Supplementary Material 3

## Data Availability

The datasets generated during and/or analysed during the current study are available from the corresponding author on reasonable request.
