# Supplementary material for: Identifying the primary tumour in patients with cancer of unknown primary (CUP) using [18F]FDG PET/CT: a systematic review and individual patient data meta-analysis
Source: Eur J Nucl Med Mol Imaging. 2024 Aug 14;52(1):225–36. doi: 10.1007/s00259-024-06860-1 (PMC11599304; doi:10.1007/s00259-024-06860-1)
Supplement: Supplementary file 1 — Supplementary Material 1 [file 259_2024_6860_MOESM1_ESM.pdf]

Online Resource 2: QUADAS-2

This supplementary information is part of *"Identifying the primary tumour in patients with cancer of unknown primary (CUP) using FDG PET/CT: a systematic review and individual patient data meta-analysis".*

Online Resource 2A: QUADAS-2 criteria

Signalling questions to assess included articles are listed below.

Risk of Bias

Patient selection:

Were consecutive or random CUP patients enrolled?

Index test

Was the interpretation of FDG PET/CT interpreted blindly by experienced nuclear medicine physicians?

Reference standard

Was the reference standard described in sufficient detail?

Flow and timing

Was there an appropriate (short) time-interval between CUP diagnosis and FDG PET/CT?

Applicability concerns

Patient selection

Were all patients real CUP?(i.e.: was the pre-FDG PET/CT work-up sufficient and extensive enough?)

Index test

Was FDG PET/CT acquisition described sufficiently and in line with-, or similar to standard whole-body FDG PET/CT investigations?

Reference standard
Is the reference standard used in the included studies similar to the one that would be used in the target population?

Online Resource 2B: QUADAS-2 scores

Results of the QUADAS-2 assessment.

Study	Risk of bias				Applicability concerns		
	Patient election	Index test	Reference standard	Flow and timing	Patient selection	Index test	Reference standard
Ambrosini	☺	☺	☺	☺	☺	☺	☺
Bicakci	☺	?	?	?	☺	?	?
Budak	?	?	☺	?	☺	☺	☺
Cengiz	☺	☹	☺	☺	?	☺	☺
Deonarine	☺	?	?	?	☺	☺	☺
Fencl	☹	☺	?	?	?	☺	☺
Gutzeit	☺	☺	☺	?	☺	☺	☺
Jain	☺	?	☺	☺	☺	☺	☺
Koc	?	?	?	?	☺	?	☺
Lawrence	☹	?	?	?	☹	?	?
Lee	☺	?	☺	?	☹	☺	☺
Li	☺	☺	☺	?	?	☺	☺
Mohamed	☺	☹	☺	☺	?	☺	☺
Nanni	☺	☺	☺	☺	☺	☺	☺
Nikolova	?	?	☺	?	☺	☺	☺
Ora	☺	☺	☺	?	?	☺	☺
Ozkan	☺	☺	?	☹	☹	☺	?
Park, JS	?	?	?	?	☺	☺	?
Park, SB	?	☺	☺	?	☺	☺	☺

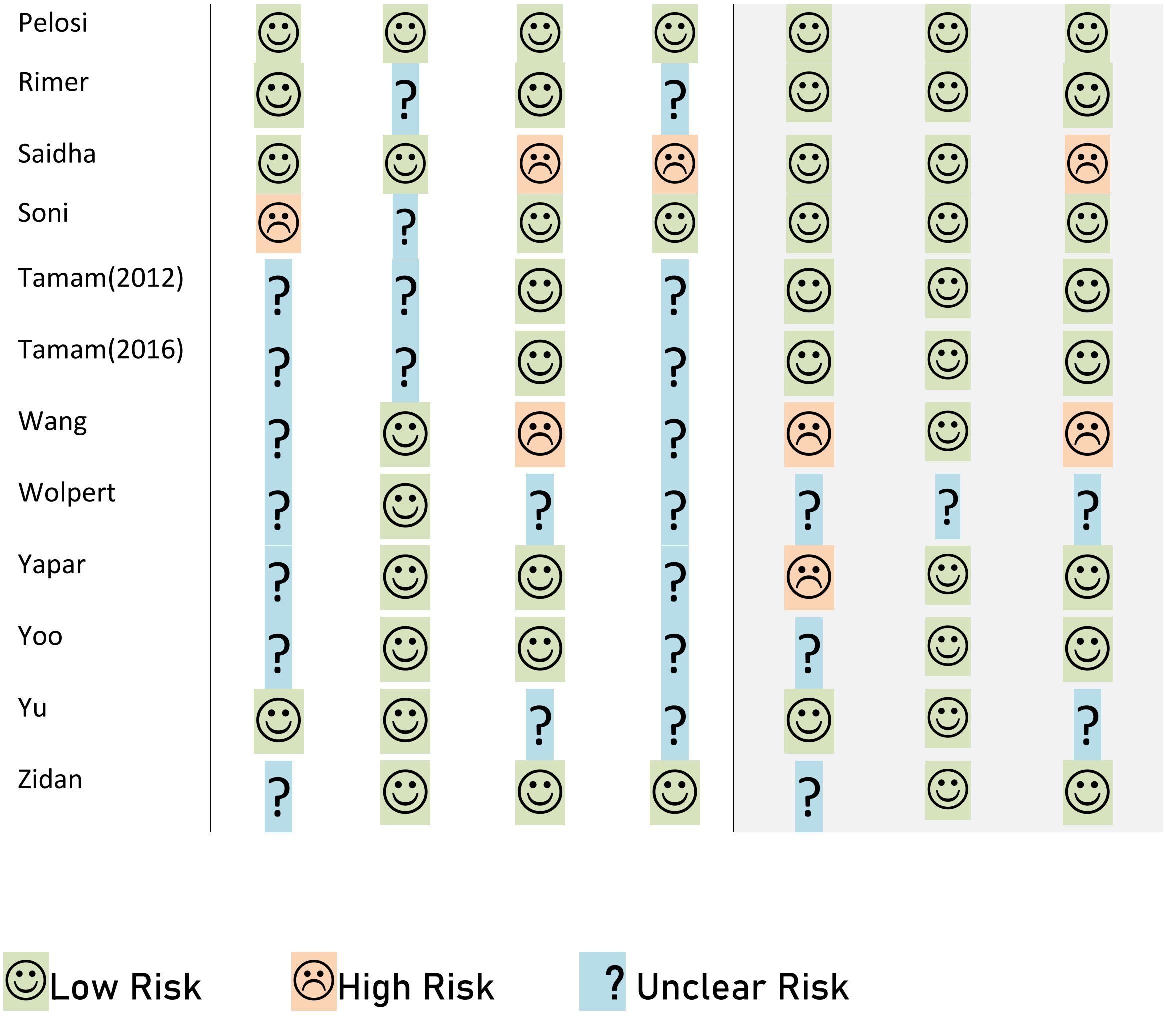

Article Title:

Identifying the primary tumour in patients with cancer of unknown primary (CUP) using FDG PET/CT: a systematic review and individual patient data meta-analysis

Journal:

European Journal of Nuclear Medicine and Molecular Imaging

Corresponding Author Details:

Max J. Lahaye, M.D., Ph.D

Department of Radiology, the Netherlands Cancer Institute

P.O. Box 90203, 1006 BE Amsterdam, The Netherlands

email: mj.lahaye@gmail.com ; m.lahaye@nki.nl

ORCID: 0000-0002-8444-202X
