# Supplementary material for: Identifying the primary tumour in patients with cancer of unknown primary (CUP) using [18F]FDG PET/CT: a systematic review and individual patient data meta-analysis
Source: Eur J Nucl Med Mol Imaging. 2024 Aug 14;52(1):225–36. doi: 10.1007/s00259-024-06860-1 (PMC11599304; doi:10.1007/s00259-024-06860-1)
Supplement: Supplementary file 2 — Supplementary Material 2 [file 259_2024_6860_MOESM2_ESM.pdf]

Online Resource 3: Grouped metastatic sites and corresponding primary tumours and classifications.

This supplementary information is part of *"Identifying the primary tumour in patients with cancer of unknown primary (CUP) using FDG PET/CT: a systematic review and individual patient data meta-analysis".*

Detailed Information on FDG PET/CT results. True primary tumours, or the final diagnosis is shown for each subgroup.

Metastatic site	True positives	False positives*	False negatives*	Definitive CUP
Bone<br>(n = 622)	*Lung/bronchial: 197*<br>*Prostate: 34*<br>*Upper GI: 18*<br>*Thyroid: 10*<br>*Testis: 1*<br>*Small bowel: 1*<br>*Sarcoma: 3*<br>*Other: 2*<br>*Mediastinum: 1*<br>*Liver: 1*<br>*Kidney/urology: 13*<br>*Head & neck: 3*<br>*Gynecology: 4*<br>*Colorectum: 13*<br>*Breast: 19*<br>*Bone: 8*<br>*Bile duct: 13*<br>Total: 348	*Bone: 4*<br>*Bile duct: 1*<br>*Breast: 2*<br>*Gynaecology: 1*<br>*Kidney/urology; 1*<br>*Lung/bronchial: 3*<br>*Prostate: 2*<br>*Upper GI: 1*<br>*Other:2*<br>*Colorectum: 1*<br>*Unknown: 20*<br>Total: 37	*Bone: 10*<br>*Lung/bronchial: 16*<br>*Lymphoma: 5*<br>*Prostate: 14*<br>*Upper GI: 5*<br>*Thyroid: 1*<br>*Sarcoma: 3*<br>*Other: 2*<br>*Multiple myeloma: 8*<br>*Colorectum: 3*<br>*Kidney/urology:2*<br>*Melanoma: 1*<br>*Breast: 5*<br>*Liver: 1*<br>*Bile duct: 1*<br>Total: 77	Total: 160
Brain<br>(n = 316)	*Lung/bronchial: 187*<br>*Brain: 12*<br>*Head & neck: 3*<br>*Other: 2*<br>*Colorectum: 6*<br>*Kidney/urology: 7*<br>*Gynaecology: 1*<br>*Liver:1*<br>*Lymphoma: 7*<br>*Mediastinum: 1*<br>*Melanoma: 3*	*Gynecology: 1*<br>*Lung/bronchial: 2*<br>*Unknown: 8*<br>Total: 11	*Brain: 1*<br>*Lung/bronchial: 4*<br>*Melanoma: 1*<br>Total: 6	Total: 53

	Pancreas: 2<br>*Prostate: 1*<br>*Skin: 1*<br>*Small bowel: 1*<br>*Thyroid: 2*<br>*Upper GI: 8*<br>*Breast: 3*<br>Total: 246			
Liver<br>(n = 369)	Appendix: 3<br>Bile duct: 6<br>Breast: 9<br>Colorectum: 47<br>Other: 11<br>Gynecology: 15<br>Head & neck: 2<br>Kidney/urology: 2<br>Liver: 15<br>Lung/bronchial: 51<br>Pancreas: 32<br>Pleura: 2<br>Prostate: 1<br>Small bowel: 5<br>Upper GI: 31<br>Total: 232	*Adrenal: 1*<br>*Bile duct: 2*<br>*Colorectum: 4*<br>*Liver: 2*<br>*Lung/bronchial: 3*<br>*Small bowel: 1*<br>*Upper GI: 2*<br>*Unknown: 8*<br>Total: 23	*Bile duct: 4*<br>*Breast: 2*<br>*Colorectum: 5*<br>*Gynecology: 1*<br>*Kidney/urology: 3*<br>*Lung/bronchial: 2*<br>*Melanoma: 1*<br>*Other: 2*<br>*Pancreas: 3*<br>*Prostate: 2*<br>*Small bowel: 1*<br>*Upper GI: 3*<br>Total: 29	Total: 85
TLN<br>(n = 164)	*Breast: 14*<br>*Gynecology: 2*<br>*Head & neck: 1*<br>*Leukemia: 1*<br>*Lung/bronchial: 28*<br>*Lymphoma: 6*<br>*Mediastinum: 1*<br>*Melanoma: 2*<br>*Multiple myeloma: 1*<br>*Other: 1*<br>*Pancreas: 3*<br>*Prostate: 1*<br>*Skin: 1*<br>*Thyroid: 2*<br>Total: 64	*Bone: 1*<br>*Unknown: 3*<br>Total: 4	*Breast: 9*<br>*Lung/bronchial: 5*<br>*Mediastinum: 1*<br>*Melanoma: 1*<br>*Other: 4*<br>*Testis: 1*<br>Total: 21	*Total: 75*
ALN<br>(n = 102)	*Bile duct: 1*<br>*Breast: 1*<br>*Colorectum: 3*<br>*Gynecology: 2*<br>*Head & neck: 2*<br>*Lung/bronchial: 4*<br>*Lymphoma: 5*<br>*Other: 2*<br>*Pancreas: 4*	*Lymphoma: 3*<br>*Unknown: 3*<br>Total: 6	*Adrenal: 1*<br>*Bile duct: 1*<br>*Gynecology: 1*<br>*Lung/bronchial: 1*<br>*Other: 3*<br>*Pancreas: 2*<br>*Lymphoma: 1*<br>Total: 10	*Total: 52*

	Penis: 1<br>*Prostate: 2*<br>*Sarcoma: 1*<br>*Small bowel: 2*<br>*Testis: 2*<br>*Upper GI: 2*<br>Total: 34			
LN(NS)<br>(n =92)	*Bile duct: 1*<br>*Colorectum: 1*<br>*Gynecology: 9*<br>*Lung/bronchial: 10*<br>*Lymphoma: 4*<br>*Head & Neck: 2*<br>*Kidney/urology: 2*<br>*Pancreas: 2*<br>*Prostate: 1*<br>*Testis: 1*<br>*Breast: 1*<br>*Thyroid: 1*<br>*Upper GI: 3*<br>*Other: 2*<br>Total: 40	*Gynecology: 3*<br>*Colorectum: 1*<br>*Kidney/urology: 1*<br>*Testis: 2*<br>*Lung/bronchial: 1*<br>*Unknown: 12*<br>Total: 20	*Melanoma: 2*<br>*Breast: 1*<br>*Penis: 1*<br>Total: 4	Total: 28
Lung<br>(n = 67)	*Bile duct: 1*<br>*Colorectum: 5*<br>*Other: 1*<br>*Gynecology: 1*<br>*Head & neck: 2*<br>*Liver: 2*<br>*Lung/bronchial: 18*<br>*Pancreas: 2*<br>Total: 32	*Unknown: 6*<br>*Total: 6*	*Gynecology: 2*<br>*Other: 1*<br>*Pancreas: 1*<br>*Lung/bronchial: 3*<br>*Kidney/urology: 1*<br>*Melanoma: 1*<br>*Prostate: 1*<br>Total: 10	*Total: 19*
Peritoneum<br>(n = 70)	*Appendix: 1*<br>*Bile duct: 4*<br>*Colorectum: 7*<br>*Gynecology: 6*<br>*Upper GI: 5*<br>Total: 23	*Unknown: 1*<br>Total: 1	*Appendix: 1*<br>*Bile duct: 1*<br>*Gynecology: 1*<br>*Other: 1*<br>*Upper GI: 1*<br>Total: 5	*Total: 41*
Soft tissue<br>(n = 23)	*Breast: 1*<br>*Kidney/urology: 2*<br>*Lung/bronchial: 3*<br>Total: 6	*Unknown: 2*<br>*Skin: 1*<br>Total: 3	*Other: 2*<br>*Breast: 1*<br>*Gynaecology: 1*<br>Total: 4	*Total: 10*

*True primary shown

Article Title:

Identifying the primary tumour in patients with cancer of unknown primary (CUP) using FDG PET/CT: a systematic review and individual patient data meta-analysis

Journal:

European Journal of Nuclear Medicine and Molecular Imaging

Corresponding Author Details:

Max J. Lahaye, M.D., Ph.D

Department of Radiology, the Netherlands Cancer Institute

P.O. Box 90203, 1006 BE Amsterdam, The Netherlands

email: mj.lahaye@gmail.com ; m.lahaye@nki.nl

ORCID: 0000-0002-8444-202X
