# Supplementary material for: Identifying the primary tumour in patients with cancer of unknown primary (CUP) using [18F]FDG PET/CT: a systematic review and individual patient data meta-analysis
Source: Eur J Nucl Med Mol Imaging. 2024 Aug 14;52(1):225–36. doi: 10.1007/s00259-024-06860-1 (PMC11599304; doi:10.1007/s00259-024-06860-1)
Supplement: Supplementary file 3 — Supplementary Material 3 [file 259_2024_6860_MOESM3_ESM.pdf]

Online Resource 1: Search strategy

This supplementary information is part of *"Identifying the primary tumour in patients with cancer of unknown primary (CUP) using FDG PET/CT: a systematic review and individual patient data meta-analysis".*

Ovid MEDLINE(R) ALL <1946 to February 14, 2024>		
1	(exp Deoxyglucose/ or ("deoxyglucose" or "desoxyglucose" or "deoxy-glucose" or "desoxy-glucose" or "deoxy-d-glucose" or "desoxy-d-glucose" or "2deoxyglucose" or "2deoxy-d-glucose" or "fluorodeoxyglucose" or "fluorodesoxyglucose" or "fludeoxyglucose" or "fluordeoxyglucose" or "fluordesoxyglucose" or "18fluorodeoxyglucose" or "18fluorodesoxyglucose" or "18fluordeoxyglucose" or "fdg" or "18fdg*" or "18f-dg*" or "18f-fdg*" or "fdg18*" or "fdgpet*").ti,ab,kf. or (("fluor" or "2fluor*" or "fluoro" or "fluorodeoxy" or "fludeoxy" or "fluorine" or "18f" or "18flu*") and ("glucose" or "galactose")).ti,ab,kf.) and (exp Tomography, Emission-Computed/ or ("pet" or "pet/ct" or "petct" or "petscan*" or "fdgpet*").ti,ab,kf. or ("emission" and "tomogra*").ti,ab,kf.)	57083
2	exp Neoplasms, Unknown Primary/ or (((unknown or occult or indetermina* or hidden or obscure or discern) adj3 (neoplas* or tumor* or tumour* or cancer* or malign* or metast* or carcinoma* or adenocarc*)) or cup or (("occult primar*" or "unknown primary" or "unknown origin") and (neoplas* or tumor* or tumour* or cancer* or malign* or metast* or carcinoma* or adenocarc*))).ti,ab,kf.	56350
3	1 and 2	1319

Embase

Search Queries		
**No.**	**Query**	**Results**
#1	'cancer of unknown primary site'/exp OR 'cancer of unknown primary site' OR ((unknown:ti,ab,kw OR occult:ti,ab,kw OR indetermina*:ti,ab,kw OR hidden:ti,ab,kw OR obscure:ti,ab,kw OR discern:ti,ab,kw) AND adj3:ti,ab,kw AND (neoplas*:ti,ab,kw OR tumor*:ti,ab,kw OR tumour*:ti,ab,kw OR cancer*:ti,ab,kw OR malign*:ti,ab,kw OR metast*:ti,ab,kw OR carcinoma*:ti,ab,kw OR adenocarc*:ti,ab,kw)) OR cup:ti,ab,kw OR (('occult primar*':ti,ab,kw OR 'unknown primary':ti,ab,kw OR 'unknown origin':ti,ab,kw) AND (neoplas*:ti,ab,kw OR tumor*:ti,ab,kw OR tumour*:ti,ab,kw OR cancer*:ti,ab,kw OR malign*:ti,ab,kw OR metast*:ti,ab,kw OR carcinoma*:ti,ab,kw OR adenocarc*:ti,ab,kw))	43787
#2	('deoxyglucose'/exp OR 'deoxyglucose':ti,ab,kw OR 'desoxyglucose':ti,ab,kw OR 'deoxy-glucose':ti,ab,kw OR 'desoxy-glucose':ti,ab,kw OR 'deoxy-d-glucose':ti,ab,kw OR 'desoxy-d-glucose':ti,ab,kw OR '2deoxyglucose':ti,ab,kw OR '2deoxy-d-glucose':ti,ab,kw OR 'fluorodeoxyglucose':ti,ab,kw OR 'fluorodesoxyglucose':ti,ab,kw OR 'fludeoxyglucose':ti,ab,kw OR 'fluordeoxyglucose':ti,ab,kw OR 'fluordesoxyglucose':ti,ab,kw OR '18fluorodeoxyglucose':ti,ab,kw OR '18fluorodesoxyglucose':ti,ab,kw OR '18fluordeoxyglucose':ti,ab,kw OR 'fdg':ti,ab,kw OR '18fdg*':ti,ab,kw OR '18f-dg*':ti,ab,kw OR '18f-fdg*':ti,ab,kw OR 'fdg18*':ti,ab,kw OR 'fdgpet*':ti,ab,kw OR (('fluor':ti,ab,kw OR '2fluor*':ti,ab,kw OR 'fluoro':ti,ab,kw OR 'fluorodeoxy':ti,ab,kw	90874

	OR 'fludeoxy':ti,ab,kw OR 'fluorine':ti,ab,kw OR '18f':ti,ab,kw OR '18flu*':ti,ab,kw) AND ('glucose':ti,ab,kw OR 'galactose':ti,ab,kw))) AND ('computer assisted emission tomography'/exp OR 'pet':ti,ab,kw OR 'pet/ct':ti,ab,kw OR 'petct':ti,ab,kw OR 'petscan*':ti,ab,kw OR 'fdgpet*':ti,ab,kw OR ('emission':ti,ab,kw AND 'tomogra*':ti,ab,kw))	
#3	('cancer of unknown primary site'/exp OR 'cancer of unknown primary site' OR ((unknown:ti,ab,kw OR occult:ti,ab,kw OR indetermina*:ti,ab,kw OR hidden:ti,ab,kw OR obscure:ti,ab,kw OR discern:ti,ab,kw) AND adj3:ti,ab,kw AND (neoplas*:ti,ab,kw OR tumor*:ti,ab,kw OR tumour*:ti,ab,kw OR cancer*:ti,ab,kw OR malign*:ti,ab,kw OR metast*:ti,ab,kw OR carcinoma*:ti,ab,kw OR adenocarc*:ti,ab,kw)) OR cup:ti,ab,kw OR (('occult primar*':ti,ab,kw OR 'unknown primary':ti,ab,kw OR 'unknown origin':ti,ab,kw) AND (neoplas*:ti,ab,kw OR tumor*:ti,ab,kw OR tumour*:ti,ab,kw OR cancer*:ti,ab,kw OR malign*:ti,ab,kw OR metast*:ti,ab,kw OR carcinoma*:ti,ab,kw OR adenocarc*:ti,ab,kw))) AND (('deoxyglucose'/exp OR 'deoxyglucose':ti,ab,kw OR 'desoxyglucose':ti,ab,kw OR 'deoxy-glucose':ti,ab,kw OR 'desoxy-glucose':ti,ab,kw OR 'deoxy-d-glucose':ti,ab,kw OR 'desoxy-d-glucose':ti,ab,kw OR '2deoxyglucose':ti,ab,kw OR '2deoxy-d-glucose':ti,ab,kw OR 'fluorodeoxyglucose':ti,ab,kw OR 'fluorodesoxyglucose':ti,ab,kw OR 'fludeoxyglucose':ti,ab,kw OR 'fluordeoxyglucose':ti,ab,kw OR 'fluordesoxyglucose':ti,ab,kw OR '18fluorodeoxyglucose':ti,ab,kw OR '18fluorodesoxyglucose':ti,ab,kw OR '18fluordeoxyglucose':ti,ab,kw OR 'fdg':ti,ab,kw OR '18fdg*':ti,ab,kw OR '18f-dg*':ti,ab,kw OR '18f-fdg*':ti,ab,kw OR 'fdg18*':ti,ab,kw OR 'fdgpet*':ti,ab,kw OR (('fluor':ti,ab,kw OR '2fluor*':ti,ab,kw OR 'fluoro':ti,ab,kw OR 'fluorodeoxy':ti,ab,kw OR 'fludeoxy':ti,ab,kw OR 'fluorine':ti,ab,kw OR '18f':ti,ab,kw OR '18flu*':ti,ab,kw) AND ('glucose':ti,ab,kw OR 'galactose':ti,ab,kw))) AND ('computer assisted emission tomography'/exp OR 'pet':ti,ab,kw OR 'pet/ct':ti,ab,kw OR 'petct':ti,ab,kw OR 'petscan*':ti,ab,kw OR 'fdgpet*':ti,ab,kw OR ('emission':ti,ab,kw AND 'tomogra*':ti,ab,kw)))	1392

Scopus

((AUTHKEY (((unknown OR occult OR indetermina* OR hidden OR obscure OR discern) W/3 (neoplas* OR tumor* OR tumour* OR cancer* OR malign* OR metast* OR carcinoma* OR adenocarc*)) OR cup OR (("occult primar*" OR "unknown primary" OR "unknown origin") AND (neoplas* OR tumor* OR tumour* OR cancer* OR malign* OR metast* OR carcinoma* OR adenocarc*)))) AND (AUTHKEY ((deoxyglucose OR desoxyglucose OR deoxy-glucose OR {desoxy-glucose} OR {desoxy glucose} OR {deoxy-d-glucose} OR {deoxy d glucose} OR {deoxy-d glucose} OR {desoxy-d-glucose} OR {desoxy-d glucose} OR {desoxy d glucose} OR 2deoxyglucose OR {2deoxy-d-glucose} OR {2deoxy d glucose} OR {2deoxy-d glucose} OR fluorodeoxyglucose OR fluorodesoxyglucose OR fludeoxyglucose OR fluordeoxyglucose OR fluordesoxyglucose OR 18fluorodeoxyglucose OR 18fluorodesoxyglucose OR 18fluordeoxyglucose OR fdg OR 18fdg* OR {18f-dg*} OR {18f dg*} OR {18f-fdg} OR {18f fdg} OR fdg18* OR fdgpet* OR ((fluor OR 2fluor* OR fluoro OR fluorodeoxy OR fludeoxy OR fluorine OR 18f OR 18flu*) AND (glucose OR galactose))) AND ((pet OR {pet/ct} OR petct OR petscan* OR fdgpet*) OR (emission AND tomogra*))))) OR ((TITLE-ABS (((unknown OR occult OR indetermina* OR hidden OR obscure OR discern) W/3 (neoplas* OR tumor* OR tumour* OR cancer* OR malign* OR metast* OR carcinoma* OR adenocarc*)) OR cup OR (("occult primar*" OR "unknown primary" OR "unknown origin") AND (neoplas* OR tumor* OR tumour* OR cancer* OR malign* OR metast* OR carcinoma* OR adenocarc*)))) AND (TITLE-ABS ((

deoxyglucose OR desoxyglucose OR deoxy-glucose OR {desoxy-glucose} OR {desoxy glucose} OR {deoxy-d-glucose} OR {deoxy d glucose} OR {deoxy-d glucose} OR {desoxy-d-glucose} OR {desoxy-d glucose} OR {desoxy d glucose} OR 2deoxyglucose OR {2deoxy-d-glucose} OR {2deoxy d glucose} OR {2deoxy-d glucose} OR fluorodeoxyglucose OR fluorodesoxyglucose OR fludeoxyglucose OR fluordeoxyglucose OR fluordesoxyglucose OR 18fluorodeoxyglucose OR 18fluorodesoxyglucose OR 18fluordeoxyglucose OR fdg OR 18fdg* OR {18f-dg*} OR {18f dg*} OR {18f-fdg} OR {18f fdg} OR fdg18* OR fdgpet* OR ((fluor OR 2fluor* OR fluoro OR fluorodeoxy OR fludeoxy OR fluorine OR 18f OR 18flu*) AND (glucose OR galactose))) AND ((pet OR {pet/ct} OR petct OR petscan* OR fdgpet*) OR (emission AND tomogra*)))))

Database		Number of hits before deduplication	Number of hits after deduplication
Medline (ovid)		1319	1316
Embase (ovid)		1392	747
SCOPUS		1421	222
	Total	4132	2285

Article Title:

Identifying the primary tumour in patients with cancer of unknown primary (CUP) using FDG PET/CT: a systematic review and individual patient data meta-analysis

Journal:

European Journal of Nuclear Medicine and Molecular Imaging

Corresponding Author Details:

Max J. Lahaye, M.D., Ph.D

Department of Radiology, the Netherlands Cancer Institute

P.O. Box 90203, 1006 BE Amsterdam, The Netherlands

email: mj.lahaye@gmail.com ; m.lahaye@nki.nl

ORCID: 0000-0002-8444-202X
